# News in caecal signalling: the role of propionate in microbial-epithelial crosstalk

**DOI:** 10.1007/s00424-021-02579-2

**Published:** 2021-05-24

**Authors:** Friederike Stumpff, David Manneck, Holger Martens

**Affiliations:** Institute of Veterinary Physiology, Department of Veterinary Medicine, Freie Universität Berlin, Oertzenweg 19b, 14163 Berlin, Germany

When the digesta pass from the small to the large intestine via the caecum, a dramatic change takes place. Bacterial counts surge from below 10^4^ CFU·mL^−1^ to levels above 10^11^ CFU·mL^−1^, the highest level of microorganisms found in any ecosystem [9]. Since microbiota are extremely flexible in degrading whatever energy-rich material has escaped the digestive processes of the small intestine, one advantage of this arrangement is to allow the host to extract usable calories from otherwise indigestible polysaccharides [13]. Due to the anaerobic milieu in caecum and colon, carbohydrate breakdown is halted at the level of short chain fatty acids (SCFA), which are absorbed and utilized by the host as a source of energy or to synthesize more complex carbohydrates. On the downside, an imbalance in the gut microbial community can trigger immunological responses that range from simple diarrhea to chronical inflammatory bowel disease [1] via pathways that are currently very poorly understood. There is considerable evidence to suggest that the shift from traditional, poorly digestible plant-based foods to refined sugar and animal fat plays a major role in the current world-wide epidemic of both Crohn’s disease and colitis ulcerosa, highlighting the complex interplay between dietary, microbial, immunological and genetic factors that lead to either health or disease in individuals [5, 12].

The caecum plays a central role in ensuring that the digesta flowing into the hindgut are inoculated with an optimal bacterial flora [14]. A minimal requirement for the survival of this flora is keeping luminal pH in a range around 6.5 [6]. This is no small task considering that SCFA rise to levels over 100 mmol·L^−1^ in the caecum, releasing an almost equimolar amount of protons. Buffering is urgent and must be fine-tuned to reflect the requirements of the moment, which vary with the influx and composition of the digesta. However, very little is currently known about the signalling that makes this possible.

One of the substrates produced by microbials in the fermentational process is propionate. It has been known for some time that binding of propionate to G-protein coupled SCFA receptors (FFAR2 and FFAR3) induces a release of acetylcholine from non-neuronal cells in the caecum and colon [3, 15]. According to the established notion, acetylcholine binds to muscarinic receptors, inducing an increase in the short circuit current in Ussing chambers that — with good reason — was originally thought to reflect secretion of chloride via the traditional coupling of NKCC with CFTR. In the current issue of Pflüger’s Archiv, Ballout and Diener break with this assumption, demonstrating that at least in the caecum, a major part of the current reflects secretion of HCO_3_^−^. Basolateral expression of NBCe2B and NBCn1, which mediate electrogenic and electroneutral cotransport of Na^+^ and HCO_3_^−^, generate the driving force. That HCO_3_^−^ is a substrate of CFTR is hardly new [2, 7, 11], but just why some secretagogues primarily induce secretion of Cl^−^ via CFTR, while others primarily drive efflux of HCO_3_^−^ remains to be clarified. Both the formation of signalling complexes between receptors and transporters and differential expression by distinct cell types along the crypt-villus axis are possibilities that need to be explored. Thus, in the small intestine, a cell-specific distribution pattern of CFTR, NKCC1 and NBCe1 has been reported that can be modulated by carbachol [8].

However this may be, propionate-induced secretion of HCO_3_^−^ as demonstrated by Ballout and Diener provides additional buffering via bicarbonate precisely when microbial activity commences and levels of SCFA and protons surge. This is important for preventing a shift in the microbial flora towards an acidophile, lactate producing microbiome which would eventually damage the epithelium [6]. Furthermore, enhanced secretion of HCO_3_^−^ promotes the unfolding of mucine, which is central to protecting the epithelium from microbial damage [4, 10]. While it is clear that much work still needs to be done before the factors leading to the current surge in inflammatory bowel disease are understood [5, 12], unravelling the secrets of epithelial-microbial crosstalk in the caecum is certainly a worthwhile starting point.
